# Magneto-thermal switching using superconducting metals and alloys

**DOI:** 10.1080/14686996.2025.2506978

**Published:** 2025-05-27

**Authors:** Hiroto Arima, Takumi Murakami, Poonam Rani, Yoshikazu Mizuguchi

**Affiliations:** a National Metrology Institute of Japan, National Institute of Advanced Industrial Science and Technology, Tsukuba, Ibaraki, Japan; b Department of Physics, Tokyo Metropolitan University, Hachioji, Japan

**Keywords:** Magneto-thermal switching, thermal conductivity, superconductor, critical field, alloy, phase separation, solder

## Abstract

Superconductors exhibit low thermal conductivity (*κ*) due to the suppression of electronic thermal conduction in the superconducting states with Cooper pairs. This change in *κ* enables superconductors to function as magneto-thermal switches (MTS). In this article, we review MTS behavior in pure-metal superconductors, phase-separated superconductors, and alloy-based superconductors. A large switching ratio can be achieved using high-purity superconducting metals. Nonvolatile MTS is observed in phase-separated superconductors, where the flux-trapping states are crucial for the nonvolatile MTS.

## Impact Statement

Superconducting materials are promising for low-temperature thermal management and can achieve large magneto-thermal switching ratios. Recently, nonvolatility of magneto-thermal switching has been observed in phase-separated superconducting composites.

## GRAPHICAL ABSTRACT

**Figure:**
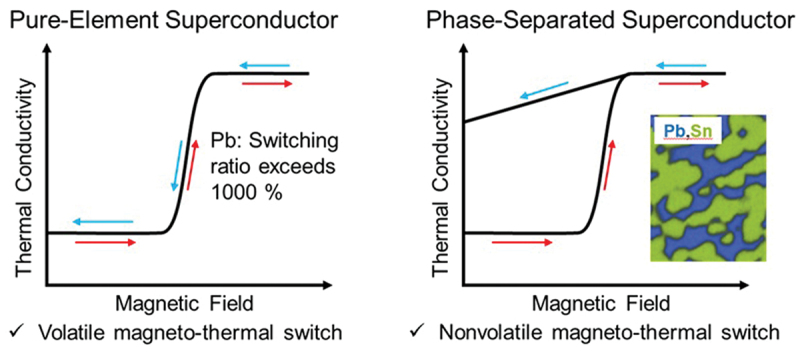

## 1. Introduction

In recent years, the miniaturization and performance enhancement of electronic devices have progressed rapidly, driven by advanced semiconductor technology [1,2]. Appropriate thermal management is essential to maintain high performance. Increased device density due to miniaturization leads to heat accumulation, and enhanced information processing capabilities result in increased heat generation [3]. Consequently, thermal management has emerged as a critical issue in various industries. Thermal management is equally important in low-temperature environments, such as in laboratories and outer space, where systems operate under cryogenic conditions. In space engineering, precise temperature regulation of various spacecraft instruments is indispensable. For instance, in Earth and astronomical observation satellites, effective thermal control, including cooling of detectors, is vital for reducing noise and achieving high measurement sensitivity [4–6].

Thermal switches are devices used in cryogenic engineering to control heat transfer, by switching thermal conduction between the two ends. In a mechanical contact thermal switch, heat conduction is controlled by physical contact or separation of materials. These switches utilize shape memory alloys [7,8], materials with different thermal expansion properties [9–12], and piezoelectric actuators [13,14]. In a gas-gap heat switch [15–17], thermal conductivity (*κ*) is turned on or off by introducing or removing gas within a sealed chamber. A third type of thermal switch operates via the application of an external field, such as an electric or magnetic field. Thermal switches based on external fields involve metal – insulator transitions, electrochemical intercalation, control of magnetic domain structures, magnons, and magneto-thermal resistance [18–25]. However, several of these materials have limited operating-temperature ranges or are not suitable for low-temperature environments.

Superconductor-based thermal switches can operate at cryogenic temperatures [26–28]. These are known as magneto-thermal switches (MTS), as their *κ* can be controlled using a magnetic field. In metals, *κ* is the sum of the electronic contribution (*κ*_el_) and the phonon contribution (*κ*_ph_). When a metal transitions into its superconducting state, Cooper pairs do not contribute to heat transport. Consequently, the electronic contribution is neglected and *κ* of the superconductor is primarily influenced by *κ*_ph_. This significantly reduces the thermal conductivity at temperatures below the superconducting transition temperature (*T*_c_). Applying a magnetic field that exceeds the critical field induces a phase transition from the superconducting state to the normal state, restoring *κ*_el_ and increasing *κ*. Therefore, superconducting MTS operate by utilizing the difference between the low conductivity in the superconducting state and the high conductivity in the normal state. These switches have practical applications in adiabatic demagnetization refrigerators (ADR). For example, the continuous ADR (CADR) developed at NASA’s Goddard Space Flight Center uses multiple cooling stages using paramagnetic materials to maintain the detector stage at temperatures as low as 50 mK [29,30]. A critical component of this system is the superconducting thermal switch that connects the coldest stage of the CADR, operating below 0.4 K.

Although numerous studies have investigated MTS in superconductors, only a few have quantitatively evaluated parameters such as the switching ratio and response speed. While some researchers have examined the process of applying a magnetic field, studies addressing the hysteresis in thermal conductivity, particularly during the reduction of the magnetic field, remain limited. In this review, we summarize our previous work, focusing on MTS in pure-element and phase-separated superconductors. For pure-element superconductors, we discuss the influence of factors such as material purity and type of superconductivity on the thermal conductivity. A notable finding from studies on phase-separated superconductors is the emergence of ‘nonvolatility of MTS’, a property not previously demonstrated in other MTS materials.

## 2. MTS in pure-element superconductors

**Figure 1.**
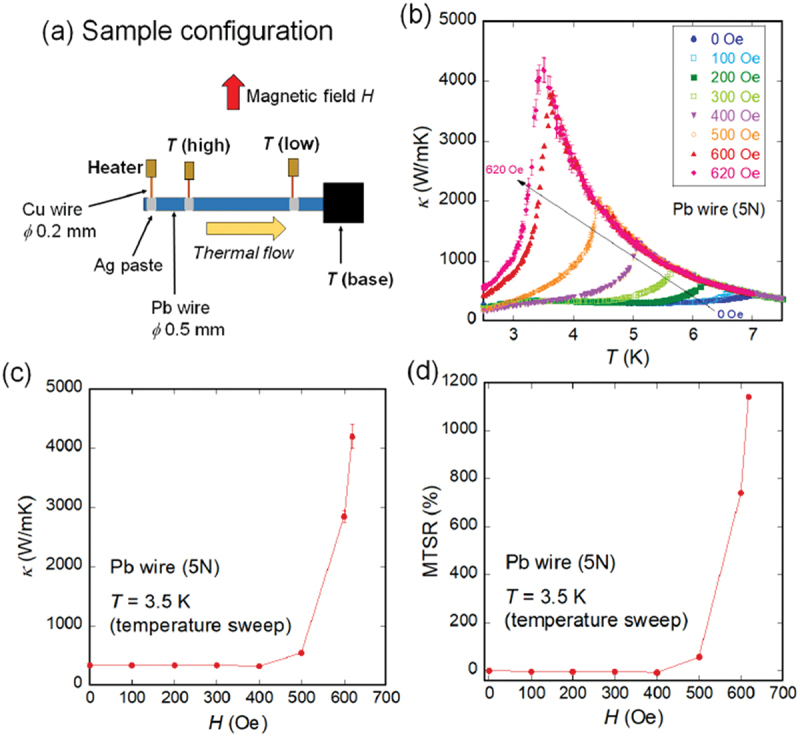
(a) Schematic of sample configuration for TTO measurements. (b) Temperature dependencies of *κ* for 5N-Pb wire at various *H*. (c,d) *H* dependence of *κ* and MTSR. Reproduced from M. Yoshida et al., J. Appl. Phys. 134, 065102 (2023) [31], published by AIP Publishing. Permission for reuse has been obtained via the RightsLink system.

Figure 1 shows a typical example of an MTS using the superconducting transition of pure-element Pb (5N purity) [31]. We measured *κ* using thermal transport option (TTO) in Physical Property Measurement System (PPMS, Quantum Design, United States); the measurement configuration is shown in Figure 1(a). As shown in Figure 1(b), the temperature dependence of *κ* changes significantly with temperature and applied field (*H*). At zero magnetic field, *κ* decreases at approximately *T*_c_ = 7.2 K, due to the superconducting transition of Pb. When a magnetic field is applied, *T*_c_ shifts to a lower temperature. Under high magnetic fields, *κ* of normal-state Pb increases as the temperature reaches *T*_c_ and drops abruptly when the temperature falls below *T*_c_. A similar increase in *κ* is observed in high -purity metals [32]. This behavior allows Pb to function as a thermal switch by exploiting the difference in thermal conductivity in the presence and absence of a magnetic field.

By extracting *κ* values from Figure 1(b) at *T* = 3.5 K, the *H* dependence of *κ* is obtained, as shown in Figure 1(c). Sharp switching was observed near *H*_c_, where *H*_c_ is the critical field. In Figure 1(d), the magnetic thermal switching ratio (MTSR), which is defined as MTSR (*T*, *H*) = [*κ* (*T*, *H*) − *κ* (*T*, *H* = 0 Oe)]/*κ* (*T*, *H* = 0 Oe), is plotted as a function of *H*. At 3.5 K, MTSR exceeded 1000%.

**Figure 2.**
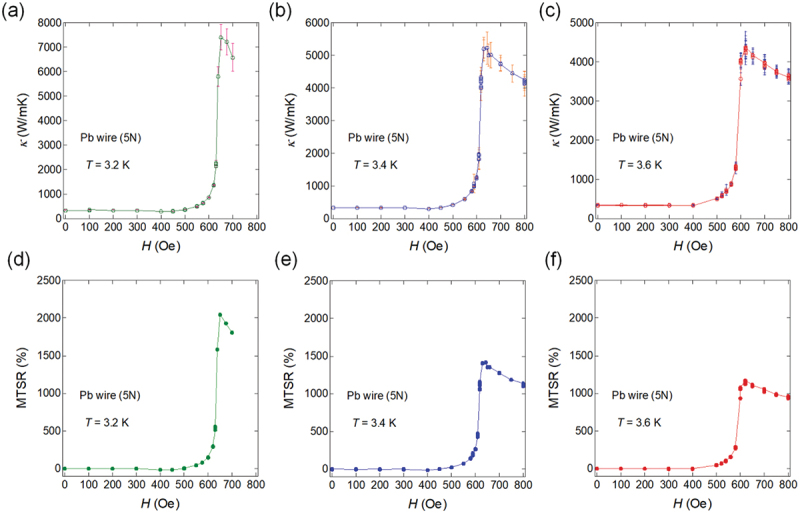
(A – c) *H* dependence of *κ* at *T* = 3.2, 3.4, and 3.6 K at the fixed temperature. (d – f) *H* dependence of MTSR at *T* = 3.2, 3.4, and 3.6 K at the fixed temperature. Reproduced from M. Yoshida et al., J. Appl. Phys. 134, 065102 (2023) [31], published by AIP Publishing. Permission for reuse has been obtained via the RightsLink system.

Figure 2(a–c) shows the magnetic field dependence of *κ* at *T* = 3.2, 3.4, and 3.6 K. These *κ* values are obtained by changing the magnetic field while temperature is fixed. In the superconducting state, *κ* remains low; however, above *H*_c_, it rises sharply. The value of maximum *κ* above *H*_c_ increases as the temperature decreases, reaching up to 7200 W/mK at *T* = 3.2 K. The MTSR values calculated from Figures 1(a–c) are shown in Figures 2(b–d). The maximum MTSR increased with a decreasing temperature, exceeding 2000% at *T* = 3.2 K.

As shown in Figure 2, *κ* in Pb increases sharply. This is caused by the sharp superconducting transition of type-I superconductors in which the transition from Meissner (perfect diamagnetism) states to normal states suddenly occurs at *H*_c_. By contrast, in type-II superconductors, the transition from the Meissner states to the normal states occurs gradually. At *H* = *H*_c1_ (lower critical field), type-II superconductors begin to accept magnetic fluxes inside the superconductor (mixed state), and the magnetic flux is present in a vortex form up to *H* = *H*_c2_ (upper critical field). Subsequently, at *H* > *H*_c2_, the superconducting states are perfectly suppressed.

**Figure 3.**
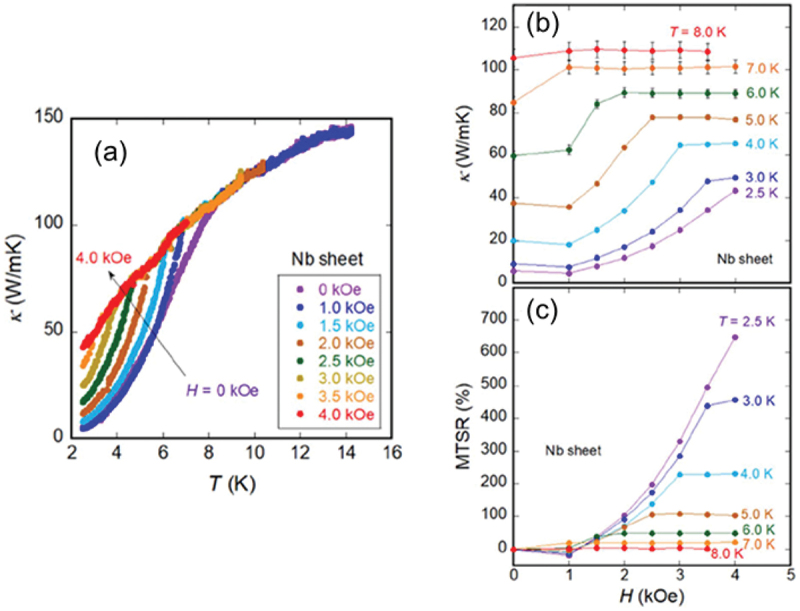
(a) Temperature dependencies of *κ* of Nb (3N) sheet at various *H*. (b,c) *H* dependencies of *κ* and MTSR determined from the *κ*–*T* data. Reproduced from M. Yoshida et al., Appl. Phys. Express 16 033002 (2023) [33] under the terms of the creative commons attribution 4.0 license.

Figure 3(a) illustrates the *H* dependence of *κ* of Nb (3N) [33]. At zero magnetic field, *κ* decreases with decreasing temperature in the normal conducting state, with a sharper decline below *T*_c_. When a magnetic field is applied, the superconducting transition temperature *T*_c_ of Nb shifts to a lower temperature. However, unlike high-purity 5N-Pb, *κ* in Nb does not exhibit a significant increase in the normal state. Figure 3(b) presents the *H* dependence of *κ* extracted from Figure 3(a). At *T* = 3.0 K, *κ* begins to increase gradually when *H* exceeds *H*_c1_ at approximately 1000 Oe and then saturates when *H* beyond *H*_c2_ at approximately 3500 Oe. By contrast, at *T* = 8.0 K, which is close to *T*_c_ of Nb, *κ* remains nearly constant across the entire magnetic field. Compared to Pb, the transition in Nb from the low-*κ* (superconducting) state to the high-*κ* (normal conducting) state is relatively broad, which is caused by the presence mixed state between *H*_c1_ and *H*_c2_. Figure 3(c) shows the MTSR calculated from Figure 3(b). At *T* = 2.5 K, the maximum MTSR approaches 700%, with its peak value decreasing as the temperature increases.

**Figure 4.**
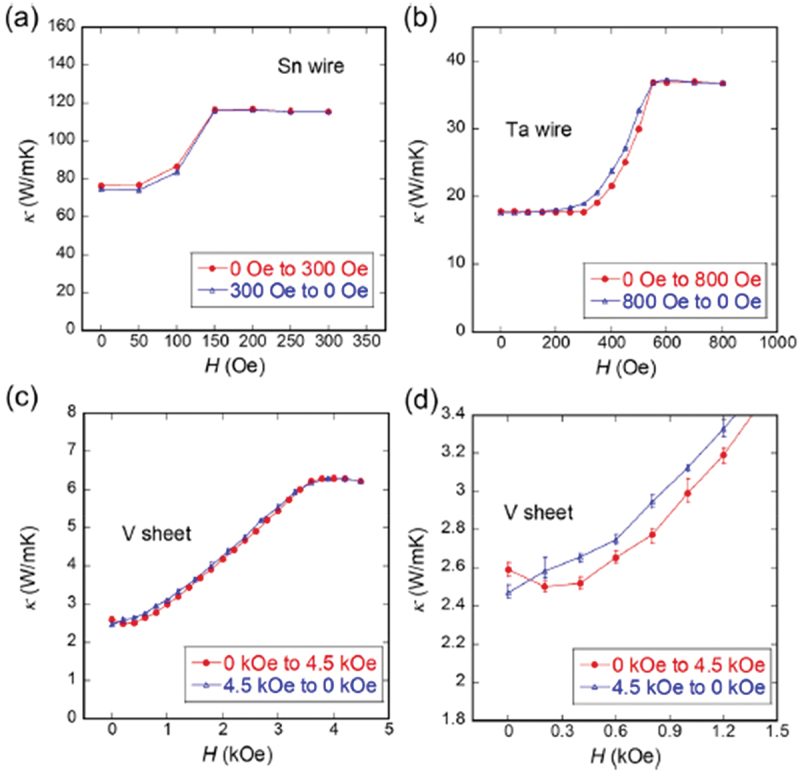
*κ*–*H* curve for (a) Sn wire (3N purity), (b) Ta wire (3N-up purity), and (c,d) V sheet (3N purity). Red and blue markers indicate increasing and decreasing field processes, respectively. Reproduced from M. Yoshida, H. Arima, Y. Watanabe, A. Yamashita, and Y. Mizuguchi, Physica C: Superconductivity and its Applications 623, 1354536 (2024) [34], published by Elsevier. Permission has been granted through the RightsLink system.

**Figure 5.**
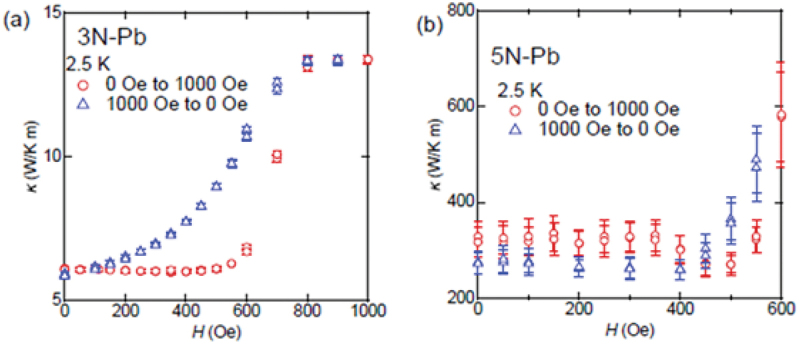
*κ*–*H* for (a) 3N-Pb wire and (b) 5N-Pb wire. Red and blue markers indicate increasing and decreasing field processes, respectively. Reproduced from H. Arima, M. Yoshida, and Y. Mizuguchi, J. Phys. Soc. Jpn. 93, 015001 (2024) [35], published by the Physical Society of Japan.

Hereafter, we focus on the magnetic field hysteresis on the *κ*–*H* plot for various superconductors. Figure 4 shows the *κ*–*H* curve for (a) Sn wire (3N purity, type-I), (b) Ta wire (3N-up purity, type-I), and (c,d) V sheet (3N purity, type-II). As shown in Figures 4(a,b), Sn and Ta exhibit weak hysteresis between the field-increasing (red markers) and the field-decreasing (blue markers) processes and the behavior of the hysteresis differs. For Sn, *κ* during the field-decreasing process is slightly lower than that during the field-increasing process and the *κ* at zero field differs depending on whether the sample experience a magnetic field. By contrast, *κ* in Ta exhibits slightly higher during the field-decreasing process and the zero-field *κ* remains unchanged before and after the application of magnetic field. The origin of this hysteresis in type-I superconductors is not yet fully understood; however, it is likely due to impurities, as has also observed in Pb (Figure 5). For the type-II superconducting V sheet shown in Figures 4(c,d) the overall trend is similar to that of Ta, where *κ* in the field-decreasing process is generally higher than that in the field-increasing process. However, the detailed field dependence exhibited differences. Specifically, as shown in Figure 4(d), *κ* in the V sheet decreases slightly from zero field up to approximately 0.3 kOe, followed by an increase at high fields. A minimum around 0.3 kOe was not observed during the field-decreasing process. This behavior is consistent with previous studies on type-II superconductors [34–36]. When the magnetic field exceeds *H*_c1_, vortex formation leads to enhanced phonon scattering, resulting in a reduction of the phonon contribution to thermal conductivity (*κ*_ph_). With further increases in the magnetic field, *κ*_el_ becomes more significant, leading to an overall increase in *κ*. The discrepancy in zero-field *κ* before and after magnetic field experience is attributed to a reduction in *κ*_ph_ caused by trapped magnetic flux and an enhancement of *κ*_el_ due to the presence of normal conducting regions.

Sn and Ta exhibit only slight hysteresis in the field dependence of *κ* and 3N-Pb shows pronounced hysteresis, as shown in Figure 5(a). X-ray fluorescence (XRF) (JEOL Ltd., Japan) analysis revealed the presence of Cu impurities in the 3N-Pb sample. In contrast, the 5N-Pb sample, shown in Figure 5(b), does not exhibit such broad hysteresis. These observations indicate that the *κ* of Pb is highly sensitive to impurity content. This pronounced hysteresis behavior observed in Pb led us to further investigate MTS properties of highly disordered superconducting alloys such as phase-separated alloys containing Pb to explore ‘*nonvolatile MTS*’.

## 3. Nonvolatile MTS in Sn–pb solders

**Figure 6.**
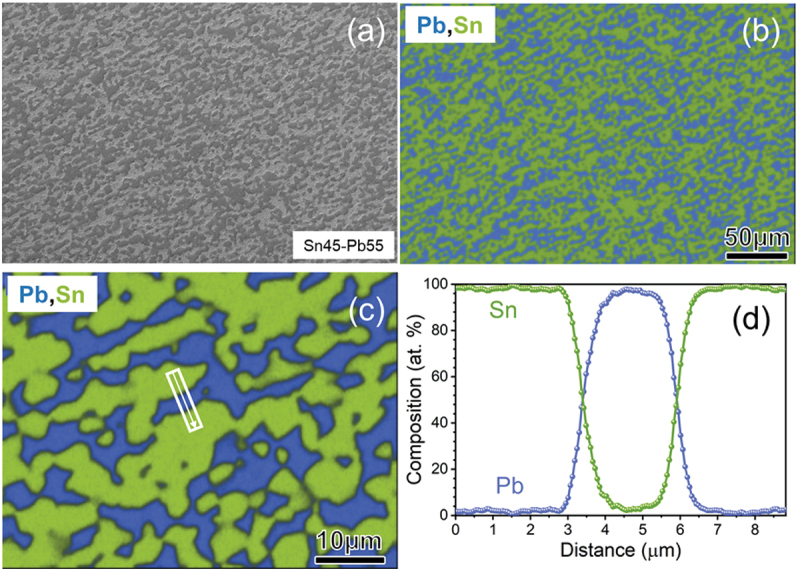
SEM – EDX analysis results for Sn45–Pb55 solder. The arrow in (c) indicates the line analysis position, and the result is shown in (d). Reproduced with permission from H. Arima, Md. R. Kasem, H. Sepehri-Amin, F. Ando, K. Uchida, Y. Kinoshita, M. Tokunaga, and Y. Mizuguchi, commun. Mater. 5, 34 (2024) [38] under the terms of the creative commons attribution 4.0 license.

As discussed above, achieving a nonvolatile MTS in pure-metal superconductors is challenging. Although phonon-contribution-driven hysteresis in *κ–H* was observed in type-II superconductors with/without vortices [37], development of controllable and large nonvolatile MTS using the changes in *κ*_el_ is required for practical application. Recently, a large MTS with nonvolatility was found in commercial Sn – Pb solders [38]. These solders are completely phase-separated composites. Figure 6 shows the scanning electron microscopy – energy dispersive X-ray spectroscopy (SEM – EDX) (Carl Zeiss, Germany) results for the Sn45–Pb55 solder (mass ratio of Sn:Pb = 45:55, flux-core-free; Taiyo Electric Industry Co., Ltd.). The distributions of Sn and Pb were random, and the line profile obtained by EDX showed the perfect phase separation of Sn and Pb, which indicated that the Sn45–Pb55 solder was composed of type-I superconductors.

**Figure 7.**
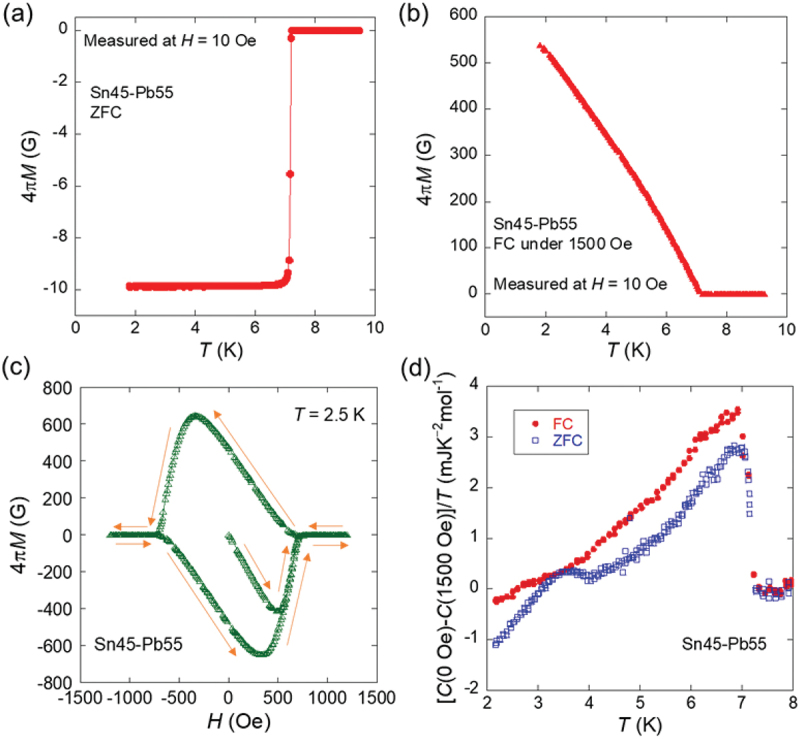
(a) Temperature dependence of magnetization (4π*M*) of Sn45–Pb55 measured after ZFC. (b) Temperature dependence of 4π*M* measured after FC under 1500 oe. (c) *H* dependence of 4π*M*. (d) Temperature dependence of (*C*(0 oe)–*C*(1500 Oe))/*T*, superconductivity contribution on specific heat (*C*) estimated by subtracting normal-state *C* from *C* in the superconducting states. Reproduced with permission from H. Arima, Md. R. Kasem, H. Sepehri-Amin, F. Ando, K. Uchida, Y. Kinoshita, M. Tokunaga, and Y. Mizuguchi, commun. Mater. 5, 34 (2024) [38] under the terms of the creative commons attribution 4.0 license.

Figures 7(a–c) present the magnetic properties of the Sn45–Pb55 solder. Figures 7(a,b) show the temperature dependence of magnetization (4π*M*) measured after zero-field cooling (ZFC) and field cooling (FC) under 1500 Oe. For ZFC, a sharp superconducting transition with perfect diamagnetism was observed for temperatures below *T*_c_ = 7.2 K. The single-step transition can be explained by the proximity effect: supercurrents flows in both the Pb and Sn regions due to the Pb superconducting states. Conversely, the magnetization measured after FC exhibited significantly positive values due to magnetic flux trapping. Figure 7(c) shows *H* dependence of 4*πM*, exhibiting hysteresis behavior associated with magnetic flux trapping. Figure 7(d) shows the temperature dependence of the electronic contribution to the specific heat (*C*) measured under ZFC and FC conditions at 1500 Oe, where *C* in the normal state was subtracted from that in the superconducting state. Under ZFC conditions, sharp peaks corresponding to the superconducting transitions of Pb and Sn were observed. Under the FC conditions, only the peak corresponding to the superconducting transition of Pb was observed, whereas the peak corresponding to the superconducting transition of Sn was absent. This indicates that the superconducting transition of Sn was suppressed by flux trapping, suggesting that the magnetic flux was preferentially trapped in the Sn region. This selective flux-trapping behavior in the Sn regions is crucial for achieving nonvolatile MTS in Sn – Pb solder alloys.

**Figure 8.**
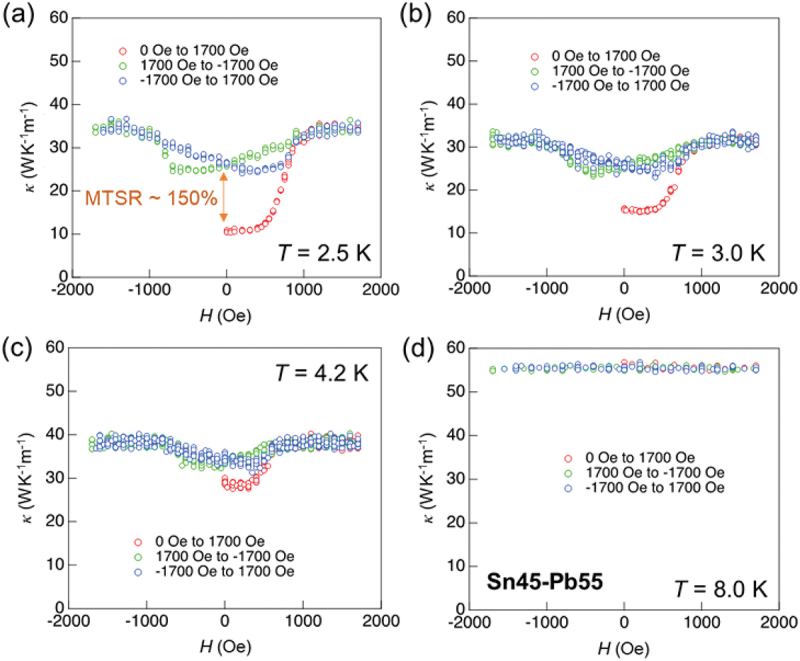
*κ*–*H* for the Sn45–Pb55 sample measured at (a) *T* = 2.5 K, (b) *T* = 3.0 K, (c) *T* = 4.2 K, and (d) *T* = 8.0 K. Reproduced with permission from H. Arima, Md. R. Kasem, H. Sepehri-Amin, F. Ando, K. Uchida, Y. Kinoshita, M. Tokunaga, and Y. Mizuguchi, commun. Mater. 5, 34 (2024) [38] under the terms of the creative commons attribution 4.0 license.

Figures 8(a–d) show the *H* dependence of *κ* imedeiately after ZFC at several temperatures. The *κ* at 2.5 K, as shown in Figure 8(a), was initially low immediately after ZFC because both Sn and Pb were in the superconducting state. Upon application of a magnetic field, *κ* increases as both Sn and Pb transition to the normal state. Notably, this high *κ* persists even after the magnetic field is reduced to zero, demonstrating the realization of a nonvolatile MTS. This nonvolatile MTS was observed below *T*_c_ of Pb, as shown in Figures 8(a–c), whereas it diminished above *T*_c_ of Pb, as shown in Figure 8(d).

The nonvolatile MTSR (NMTSR), defined as NMTSR = (*κ*(*H* = 0after *H* application)–*κ*(initial))*/κ*(initial) at 2.5 K is 150%, and NMTSR decreased with increasing temperature.

**Figure 9.**
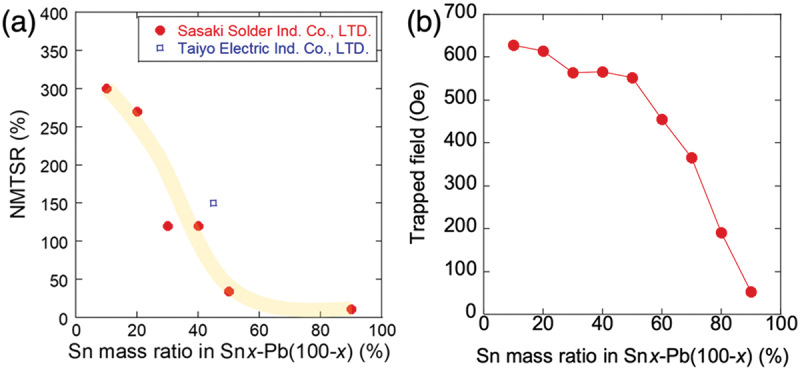
(a) Sn mass ratio (*x*) dependence of NMTSR for Sn*x*–Pb(100–*x*) solders purchased from two different companies, sasaki solder ind. Co., LTD. And taiyo electric ind. Co., LTD. Data for *x* = 10, 45, and 90 are taken from ref. 19. (b) *x* dependence of trapped field for Sn*x*–Pb(100–*x*) manufactured by sasaki solder ind. Co., LTD.

**Figure 10.**
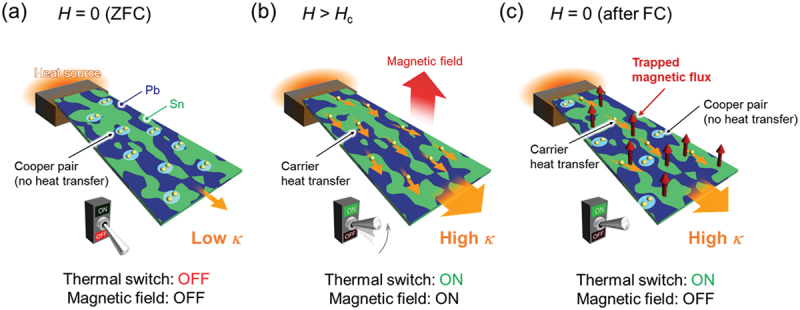
Schematic of nonvolatile MTS in Sn–pb solders. Thermal conduction at (a) *H* = 0 oe (after ZFC), (b) *H* > *H*_c_, and (c) *H* = 0 oe (after FC). Reproduced with permission from H. Arima, Md. R. Kasem, H. Sepehri-Amin, F. Ando, K. Uchida, Y. Kinoshita, M. Tokunaga, and Y. Mizuguchi, commun. Mater. 5, 34 (2024) [38] under the terms of the creative commons attribution 4.0 license.

The NMTSR is enhanced with decreasing Sn mass ratio *x* in Sn*x*–Pb(100–*x*), as shown in Figure 9. Figure S1 in supplemental materials shows the *κ*-H curves of Sn – Pb solders with different Sn masses. Although the influence of *x* on NMTSR is complex, the trend of increasing NMTSR with decreasing Sn is confirmed for products purchased from two different companies. Furthermore, the impact of Sn substitution on the NMTSR depends on the extent of magnetic flux trapping. As shown in Figure 9(b), the amount of flux trapping decreases with increasing Sn concentration. The instances of nonvolatile MTS occurring in Sn – Pb solders are summarized in Figure 10. In the Sn45–Pb55 solder, nonvolatile MTS is achieved by applying a magnetic field or FC with *H*>*H*_c_(Pb), where the superconductivity suppressed by flux trapping in the Sn region coexists with the superconducting state of Pb.

Recent studies have focused on the flux trapping in Sn – Pb solder via surface observations using a scanning superconducting quantum interference device (SQUID) microscope (SII NanoTechnology, Japan). These observations revealed that the trapped flux after FC existed in the form of quantized vortices. The presence of vortices indicated that the Sn – Pb solder contained a type-II superconducting region identified as the Sn phase [39].

## 4. Nonvolatile MTS in In–sn solders

This section summarizes the nonvolatile MTS characteristics of In – Sn solders, which are widely used as low-temperature soldering materials [40]. According to the binary phase diagram, two stable alloy phases are present: the β-phase (In-rich, *T*_c_ = 6.5 K) and the γ-phase (Sn-rich, *T*_c_  = 4.7 K) [41]. Due to the alloyed nature of these phases, the emergence of type-II superconductivity, characterized by the formation of quantized vortices, is expected in both the regions [42–45]. This behavior contrasts with that of Sn – Pb solders, which comprise both type-I and type-II superconductors.

**Figure 11.**
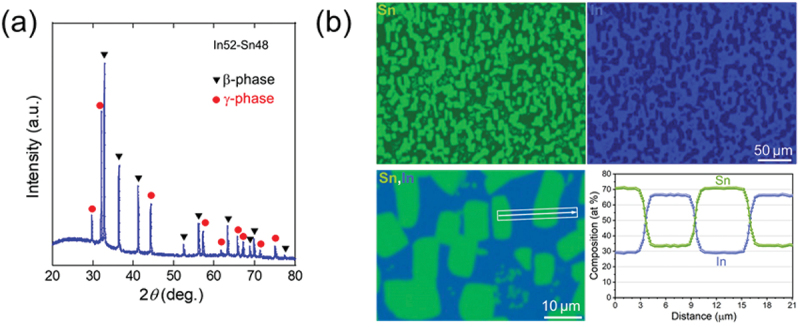
(a) XRD pattern for the In52–Sn48 solder. Black triangles indicate bragg peaks corresponding to the β-phase and red circles indicate those of the γ-phase. (b) EDX analysis results including line analysis. Reproduced with permission from P. Rani, T. Murakami, Y. Watanabe, H. Sepehri-amin, H. Arima, A. Yamashita, Y. Mizuguchi, Appl. Phys. Express 18, 033001 (2025) [40]. under the terms of the creative commons attribution 4.0 license.

Structural characterization of the In52–Sn48 solder, a commercial product from Chip Quik Inc., confirms its separation into the β- and γ- phases, as shown in the X-ray diffraction (XRD) (Rigaku, Japan) pattern in Figure 11(a). EDX mapping and line analysis (Figure 11(b)) reveal square-shaped islands corresponding to the γ-phase. The atomic concentration of In in the β-phase is approximately 66%, with the remainder consisting of Sn. In contrast, the γ-phase contains approximately 70% Sn, with the remainder consisting of In.

**Figure 12.**
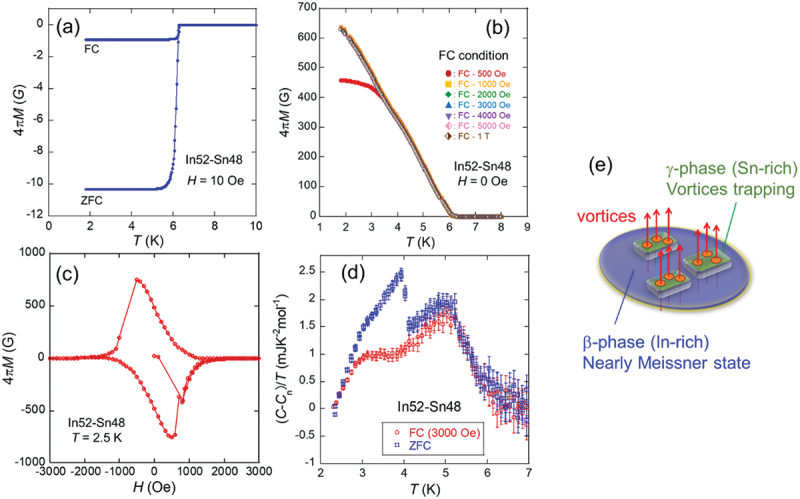
(a) Temperature dependence of magnetization (4π*M*) of In52–Sn48 measured after ZFC. (b) Temperature dependence of 4π*M* measured after FC under various fields. (c) *H* dependence of 4π*M*. (d) Temperature dependence of *C*(0 oe)–*C*(1500 Oe)/*T*. (d) Schematic of vortex trapping in the In52–Sn48 solder in the *γ*-phase. Reproduced with permission from P. Rani, T. Murakami, Y. Watanabe, H. Sepehri-amin, H. Arima, A. Yamashita, Y. Mizuguchi, Appl. Phys. Express 18, 033001 (2025) [40]. under the terms of the creative commons attribution 4.0 license.

Figure 12 illustrates the magnetization and specific heat behavior of the In52–Sn48 solder under various magnetic fields. Figure 12(a) shows the temperature dependence of magnetization (4*πM*) under ZFC and FC conditions. A superconducting transition attributed to the β-phase is observed at 6.5 K, accompanied by irreversible characteristic features of type-II superconductors in both ZFC and FC measurements. The magnetization exhibits a single-step drop at 6.5 K, with no additional change corresponding to the superconducting transition of the γ-phase. Figure 12(b) shows the temperature dependence of the magnetization after FC under various magnetic fields. For the FC at 500 Oe, approximately 470 Oe of magnetic flux was trapped at a low temperature. As the FC field increases, the trapped flux saturates above 1000 Oe, reaching approximately 630 Oe. The field dependence of magnetization at 2.5 K, as shown in Figure 12(c), clearly demonstrates irreversible hysteresis, indicative of magnetic flux trapping in the In – Sn solder after application of a magnetic field. Specific heat data shown in Figure 12(d) reveal that the superconducting transition of the β-phase remains unaffected by the FC condition, whereas the transition associated with the γ-phase is significantly suppressed. Nevertheless, an entropy change related to the γ-phase is still observed after FC, suggesting that superconductivity in the γ-phase is only partially suppressed and that magnetic vortices are present. This flux-trapping behavior is schematically illustrated in Fig. 12(e): the vortices are confined within the γ-phase regions, whereas the β-phase regions remain in a nearly ideal Meissner state.

**Figure 13.**
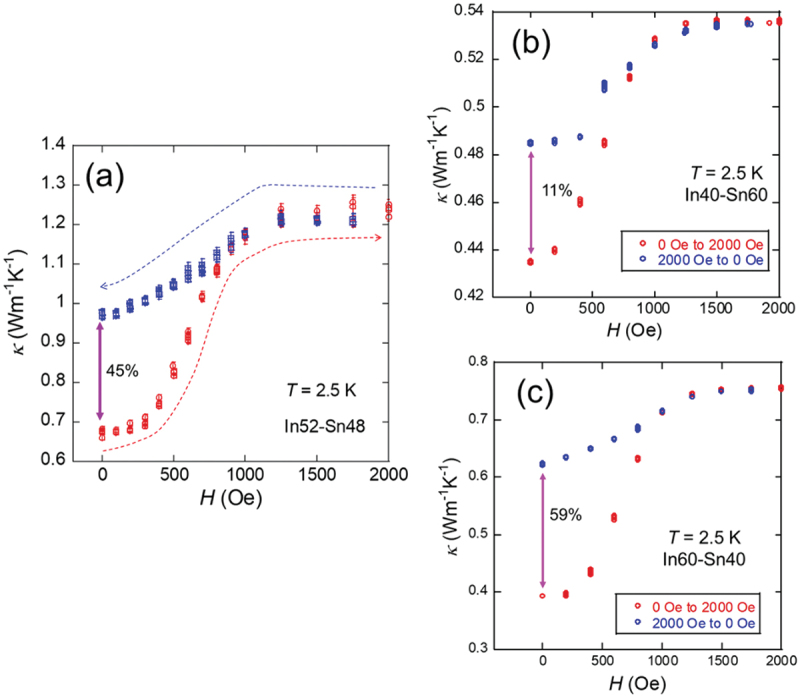
*κ*–*H* curve at *T* = 2.5 K for (a) In52–Sn48, (b) In40–Sn60, and (c) In60–Sn40. Reproduced with permission from P. Rani, T. Murakami, Y. Watanabe, H. Sepehri-amin, H. Arima, A. Yamashita, Y. Mizuguchi, Appl. Phys. Express 18, 033001 (2025) [40]. under the terms of the creative commons attribution 4.0 license.

Figure 13 shows the nonvolatile MTS properties of various In – Sn solders. In addition to the commercial In52–Sn48 solder, laboratory-prepared In – Sn solders were fabricated using a melting method in evacuated quartz tubes [41]. For In52–Sn48 solder, an NMTSR of 45% was observed, which increased to 59% for In60–Sn40. In contrast, In40–Sn60 solder exhibits a lower NMTSR of 11%. Notably, no phase separation was observed in the In20–Sn80 and In80–Sn20 samples. One key distinction between Sn – Pb and In – Sn solders is their absolute *κ* values. Sn – Pb solders are composed of pure elements such as Sn and Pb and therefore exhibit relatively high *κ*. Meanwhile, all In – Sn solders consist of one or two alloy phases, resulting in significantly lower *κ* values. A comparison of these two types of nonvolatile MTS materials suggests that the use of pure-element superconductors along with careful optimization of the island morphology, particularly with respect to the size and shape of the Sn regions, is essential for improving the nonvolatile MTS characteristics of superconductor-based systems.

## 5. Summary and future prospect

In this review, we summarized MTS studies on pure-element, phase-separated, and alloy-based superconductors. As demonstrated in high-purity Pb wires, pure metals exhibit a large *κ*_el_ at low temperatures due to minimal impurity scattering, resulting in a large switching ratio. However, the MTS behavior of pure-element superconductors is inherently volatile. To realize a nonvolatile MTS, we investigated Sn – Pb and In – Sn solders. Notably, the use of commercially available solders is a promising approach to address the challenge of realizing nonvolatile MTS. In the Sn – Pb solder system, the magnetic flux was trapped in the Sn regions. The switching between superconducting and nonsuperconducting states in these regions led to nonvolatile MTS, with the highest recorded NMTSR reaching 300% in Sn10–Pb90 at 2.5 K. In contrast, In – Sn solders consist of two alloy phases, and due to the type-II superconducting nature of these alloys, a trapped flux exists in the form of vortices. These findings indicate that a nonvolatile MTS can be realized via vortex trapping. Although the NMTSR in In–Sn solders is lower than that in Sn – Pb solders, both type-I and type-II superconductors can serve as building blocks for phase-separated, nonvolatile MTS materials. High-purity metals, typically type-I superconductors, are preferred for applications requiring large changes in thermal conductivity. Conversely, when a higher operating temperature is required, type-II superconductors, including high-*T*_c_ materials, are more suitable. Although thermal transport in various superconductors at low temperatures has been extensively studied, investigations specifically focusing on the MTS properties remain limited. To accelerate the development of nonvolatile MTS materials with improved performance, including higher NMTSR and operating temperatures, further studies on thermal transport under systematically controlled magnetic fields are essential.

## Supplementary Material

Supplemental Material
