# Supplementary material for: Magneto-thermal switching using superconducting metals and alloys
Source: Sci Technol Adv Mater. 2025 May 27;26(1):2506978. doi: 10.1080/14686996.2025.2506978 (PMC12160326; doi:10.1080/14686996.2025.2506978)
Supplement: Supplemental Material [file TSTA_A_2506978_SM1360.docx]

Supplemental materials

**Magneto-thermal switching using superconducting metals and alloys**

Hiroto Arima^1,2^, Takumi Murakami^2^, Poonam Rani^2^, Yoshikazu Mizuguchi^2^*

1. National Metrology Institute of Japan, National Institute of Advanced Industrial Science and Technology, Tsukuba, Ibaraki 305-8565, Japan
2. Department of Physics, Tokyo Metropolitan University, 1-1, Minami-osawa,

Hachioji 192-0397, Japan.


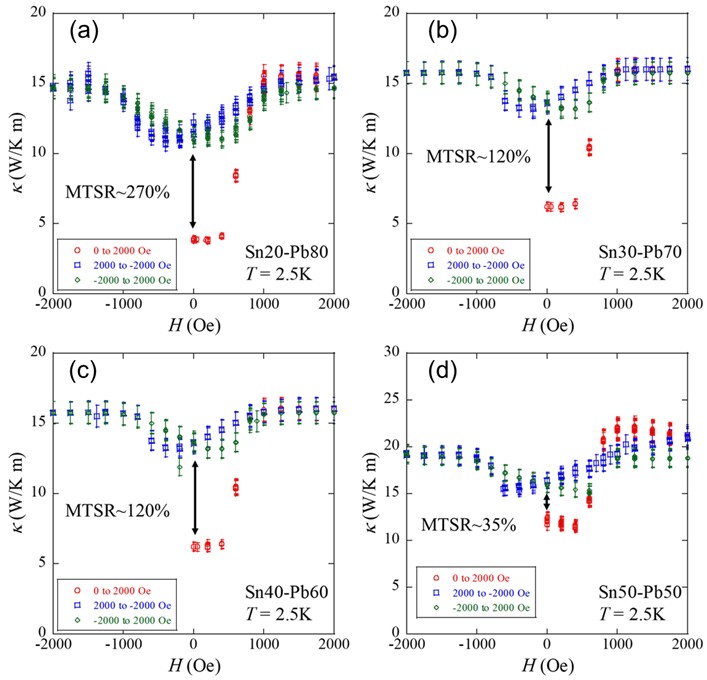


Fig. S1. Magnetic field dependence of thermal conductivity (*κ*) for (a) Sn20-Pb80, (b) Sn30-Pb70, (c) Sn40-Pb60, and (d) Sn50-Pb50.
